# Anti-MuSK-Positive Myasthenia Gravis in a Patient with Parkinsonism and Cognitive Impairment

**DOI:** 10.1155/2011/859802

**Published:** 2011-07-31

**Authors:** S. Lanfranconi, S. Corti, P. Baron, G. Conti, L. Borellini, N. Bresolin, A. Bersano

**Affiliations:** ^1^Department of Neurological Sciences, Dino Ferrari Centre, University of Milan, 20122 Milan, Italy; ^2^IRCCS Foundation Ospedale Maggiore Policlinico Mangiagalli and Regina Elena, Via F. Sforza 35, 20122 Milan, Italy

## Abstract

Muscle-specific tyrosine kinase- (MuSK-) antibodies-positive Myasthenia Gravis accounts for about one third of Seronegative Myasthenia Gravis and is clinically characterized by early onset of prominent bulbar, neck, shoulder girdle, and respiratory weakness. The response to medical therapy is generally poor. Here we report a case of late-onset MuSK-antibodies-positive Myasthenia Gravis presenting with signs of cognitive impairment and parkinsonism in addition to bulbar involvement and external ophthalmoplegia. The pattern of involvement of both peripheral and central nervous system dysfunction might suggest a common pathogenic mechanism, involving impaired cholinergic transmission.

## 1. Introduction

A generalized myasthenia gravis (MG) without detectable anti-AChR antibodies, which is commonly referred to as a seronegative Myasthenia Gravis (SNMG), is observed in about 15% of patients with generalised MG [1]. In about one third of these subjects IgG anti-Musk antibodies have been detected with a significant female predominance and earlier age at disease onset [2, 3]. Even though patients with anti-MuSK-positive MG usually develop generalized weakness at, or shortly after, onset, they display particular involvement of bulbar and facial muscles and may present with isolated neck, shoulder, or respiratory muscles weakness [4–6]. These clinical features support the idea that the absence of anti-AChR antibodies is not necessarily associated with a less severe disease.

MuSK is a transmembrane polypeptide involved in phosphorylation, clustering [7], and differentiation of postsynaptic AChR at neuromuscular junction. The underlying disease mechanism of anti-MuSK MG is still unclear. AChR levels were found to be normal in these patients [8], and it has been suggested that the presence of anti-MuSK antibodies may result in neuromuscular transmission impairment [9] probably associated with reduced miniature endplate potential amplitudes [10]. Here, we report a case of late-onset muscle-specific tyrosine kinase (MuSK)-antibodies-positive Myasthenia Gravis (MG) with bulbar weakness, external ophthalmoplegia, parkinsonism and pathologic metabolic network on cerebral FDG PET.

## 2. Case Report

A 73-years-old man was admitted to our hospital in September 2008 for a rapidly progressive bulbar dysfunction consisting of severe dysphagia, requiring a gastric tube placement, dysarthria, nasal speech, and ophthalmoplegia without ptosis. Over the past two years the patient experienced similar episodes, often less severe, with a remitting-relapsing course and spontaneous resolution within few days or weeks. In the previous three months he was hospitalized twice for severe dysphagia.

All investigations including AChR antibodies, autoimmunity and inflammatory screening but also barium swallow study, fibroscopy, and neck CT scan were normal. The clinical response to steroid therapy (methylprednisolon 1 g/d iv) for three days was inconsistent with a full recovery during the first relapse and lack of clinical improvement on the second relapse. 

At the age of 70 the patient developed asymmetrical, progressive rest tremor in his legs. Cerebral MRI performed at that time was normal. Thus, a diagnosis of atypical parkinsonism was made, and the patient was treated with levodopa-carbidopa 100/25 mg three times daily without significant improvement. 

He was referred to our centre for a further clinical evaluation. The neurological examination on admission showed masked face with decreased eye blinking and astonished look. Cranial nerve examination revealed external ophthalmoplegia with partial downward gaze sparing without dyplopia, weak forced eye closure, and severe lower cranial nerves involvement with severe dysphagia, hypophonia, nasal speech, and marked tongue weakness. Pharyngeal reflex was normal and masseter reflex quite bright. Frontal release signs such as glabellar, orbicularis oris, and palmomental reflexes were present. Asymmetrical, prominently left-sided, rest tremor was present distally in both legs. Strength was normal in the four limbs, and osteotendineal reflexes were normal except for areflexia in the left upper limb (previous trauma). Plantar response was flexor. Axial rigidity was evident together with gait disturbance characterized by tendency to turn “en bloc” and freezing. 

Biochemistry was normal including serum creatine kinase. An increased level of serum lactate was detected (2.5 mmol, n.v. 0.7–2.1 mmol), but this finding was not confirmed by a second assay (0.9 mmol). Antineuronal (Hu-Yo-Ri) antibodies were absent. Anti-AChR antibodies and voltage-gated calcium channel antibodies were also negative. Chest CT scan, spirometry, and pulmonary assessment were unremarkable.

Neurophysiological examination including complete EMG/ENG of limbs and both low-rate repetitive nerve stimulation (RNS) and single fibre EMG (SF-EMG) of the facial nerve were normal. CSF examination was normal, including CSF beta-amyloid and tau (total and phosphorylated) proteins level determination. 

**Figure 1:**
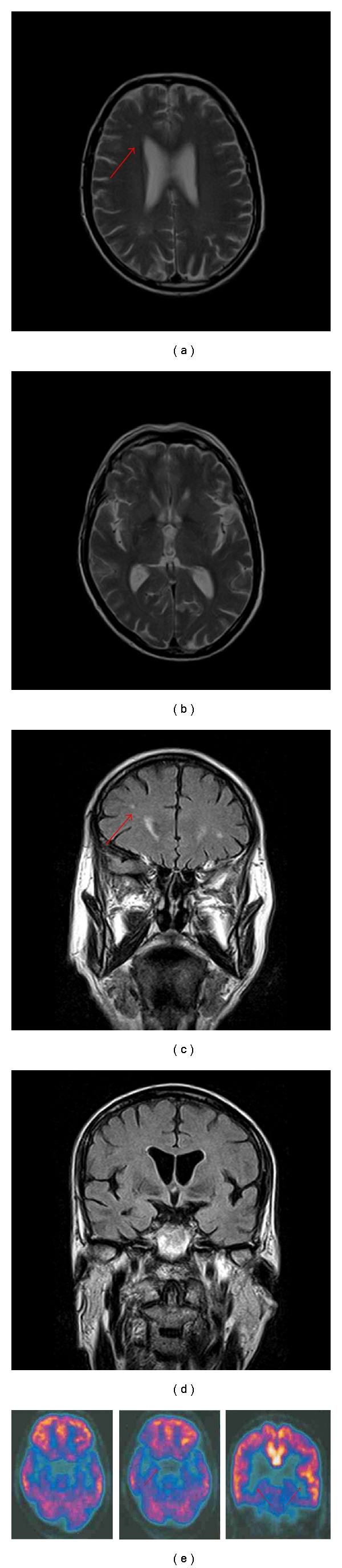
Cerebral MRI axial PD/T2weighted (a, b) and coronal FLAIR (c, d) sequences showing small bilateral aspecific white matter lesions (a, c) involving the frontal white matter (red arrows) and sparing the basal ganglia (b, d). (e) Axial and coronal cerebral FDG PET revealing a metabolic decrease in the brainstem (pons and mesencephalon) and medial temporal cortex (red arrows).

Muscle biopsy of right biceps, collected after informed consent, showed atrophy of both type I and type II muscle fibres without abnormalities of COX and SDH reactions. Cerebral MRI demonstrated slight temporal lobes atrophy and aspecific small frontal white matter lesions (Figures 1(a)–1(d)). Cerebral FDG PET revealed a metabolic decrease in the brainstem and medial temporal cortex (Figure 1(e)). Neuropsychological examination revealed a mild subcortical dementia with a predominant impairment of executive functions consisting in reduced verbal fluency, in defective planning and execution and constructive apraxia. Few days after admission, the patient presented a progressive spontaneous clinical improvement and the gastric tube was removed. He was treated with i.v methylprednisolon 1 g/d for three days followed by oral steroid assumption (prednisolon 50 mg/d) with tapering doses with mild benefit. 

Two weeks later all laboratory tests were available, and high titres of anti-MuSK (muscle-specific tyrosine kinase) antibodies were found. Thus, a diagnosis of MuSK-Ab-positive MG was established and an immunosuppressive treatment with Azathioprine 100 mg was associated with prednisolon 50 mg/d. The 30-day clinical followup revealed a full resolution of ophthalmoplegia and bulbar signs. Bradykinesia, freezing of gate, and axial rigidity remained unresponsive to L-dopa treatment.

## 3. Discussion

Early onset of prominent bulbar, neck, shoulder girdle, and respiratory weakness are the characteristic features of MuSK-antibodies-positive Myasthenia Gravis. Our case presented mild cognitive impairment and parkinsonism in association with the typical bulbar involvement and external ophthalmoplegia. A complete diagnostic workup including serum lactate determination, muscle biopsy, CSF examination, and cerebral MRI with DWI sequences excluded other differential diagnosis including Lambert Eaton syndrome, mitochondrial disorders, chronic inflammatory demyelinating polyradiculoneuropathy (CIDP), motor neuron disease and brainstem ischemia. 

Despite the fact that predominant bulbar and cranial involvement have been already described in MuSK-antibodies-positive MG [5], the clinical course of our patient was atypical. In fact, he presented with episodes of dyplopia and dysphagia worsening progressively within few days and spontaneously resolving, in absence of daily fluctuation or fatigable weakness. To our knowledge, this is the first report of MuSK-antibodies-positive MG associated to atypical parkinsonism and dysexecutive cognitive impairment. Although cranial and bulbar signs are consistent with the neuromuscular disorder, the levodopa-unresponsive parkinsonism with gait disturbance and the subcortical dementia remained without a conclusive explanation. Parkinson's disease was excluded for the absence of clinical criteria. The cerebral MRI, showing only aspecific small frontal white matter lesions, was not consistent with a vascular parkinsonism and a vascular cognitive impairment. An impaired metabolism involving the brainstem evidenced by FDG PET has been reported in PSP patients [11], but the clinical course in this patient did not satisfy clinical criteria for PSP, which require a gradually progressive course in addition to supranuclear gaze palsy and postural instability with falls [12]. 

Therefore, despite the lack of literature data on seronegative myasthenia, parkinsonism, and cognitive impairment, it might be argued whether a common mechanism might underlie the three diseases. 

Cholinergic system has been involved in the pathogenesis of movement disorders [13]. Cholinergic deficits are thought to underline gait disturbance, and cognitive impairment in PSP [14] as well as degeneration of cholinergic nuclei in the mesopontine tegmentum has been described to influence akinesia and gait initiation abnormalities in PD [15]. Finally, a reduction in nicotinic receptors was reported in neurodegenerative diseases such as AD, DLB, and PD [16–18]. Anti-Musk antibodies, other than in neuromuscular junction, were detected in brain both in neurons and nonneuronal tissues. Moreover, MuSK expression in the hippocampus seems to play a critical role in memory retention [19]. According to this finding some patients affected by MG were observed to have memory deficits [20]. 

Since anti-MuSK antibodies have been recognized to modulate AChR differentiation, phosphorylation, and clustering, a common mechanism involving an antibody response to nicotinic receptors and anti MuSK might explain both the CNS and the muscular impairment [21]. Our hypothesis is highly speculative, and further data provided by experimental models and functional studies in humans other than other case reports are necessary to support this preliminary observation.
